# Protective Effects and Mechanisms of Procyanidins on Parkinson’s Disease In Vivo and In Vitro

**DOI:** 10.3390/molecules26185558

**Published:** 2021-09-13

**Authors:** Juan Chen, Yixuan Chen, Yangfan Zheng, Jiawen Zhao, Huilin Yu, Jiajin Zhu, Duo Li

**Affiliations:** 1 Department of Food Science and Nutrition, Zhejiang University, Hangzhou 310000, China; 11613037@zju.edu.cn (J.C.); cyx901109@126.com (Y.C.); zhyfan2017@163.com (Y.Z.); 21813084@zju.edu.cn (J.Z.); yuhl0323@126.com (H.Y.); 2 Institute of Nutrition & Health, Qingdao University, Qingdao 266000, China; duoli@qdu.edu.cn

**Keywords:** procyanidins, Parkinson’s disease, PC12 cells, zebrafish, Nrf2/ARE pathway

## Abstract

This research assessed the molecular mechanism of procyanidins (PCs) against neurotoxin 1-methyl-4-phenyl-1,2,3,6-tetrahydropyridine (MPTP) and its metabolite 1-methyl-4-phenylpyridinium (MPP^+^) induced Parkinson’s disease (PD) models. In vitro, PC12 cells were incubated with PCs or deprenyl for 24 h, and then exposed to 1.5 mM MPP^+^ for 24 h. In vivo, zebrafish larvae (AB strain) 3 days post-fertilization (dpf) were incubated with deprenyl or PCs in 400 μM MPTP for 4 days. Compared with MPP^+^/MPTP alone, PCs significantly improved antioxidant activities (e.g., glutathione peroxidase (GSH-Px), superoxide dismutase (SOD), catalase (CAT)), and decreased levels of reactive oxygen species (ROS) and malondialdehyde (MDA). Furthermore, PCs significantly increased nuclear Nrf2 accumulation in PC12 cells and raised the expression of NQO1, HO-1, GCLM, and GCLC in both PC12 cells and zebrafish compared to MPP^+^/MPTP alone. The current study shows that PCs have neuroprotective effects, activate the nuclear factor-erythroid 2-related factor 2 (Nrf2)/antioxidant response element (ARE) pathway and alleviate oxidative damage in MPP^+^/MPTP-induced PD models.

## 1. Introduction

Parkinson’s disease (PD) is a progressive neurodegenerative syndrome caused by the absence of dopaminergic neurons in the substantia nigra. Its clinical manifestations include motor retardation, tremor, stiffness and postural instability [1,2,3,4,5,6]. Effective methods to prevent or reverse neuronal degeneration in PD patients are still unclear. Hence, the discovery and development of novel antiparkinsonian drugs remains a top priority in the search for PD treatments.

Although the pathogenesis of PD remains unclear, previous studies have demonstrated that oxidative stress plays a key role in the loss of dopaminergic neurons [7,8]. Dopamine can undergo auto- or enzyme-catalyzed oxidation to lead the production of reactive oxygen species (ROS) and electrophilic quinone molecules [9], both processes that may underlie the vulnerability of dopaminergic neurons to oxidative and electrophilic stress [10,11,12]. Hence, a possible method for the prevention or treatment of PD may be to supplement treatment with antioxidants to eliminate excessive ROS. Indeed, natural bioactive compounds are advantageous in that they are often safe for consumers and have very few (if any) adverse reactions [13]. Therefore, antioxidant compounds from natural resources may aid in the treatment of PD.

Procyanidins (PCs) are natural phenolic compound of flavonoids, including oligomer of monomer catechin and epicatechins [14]. PCs are natural nutrients and antioxidants with antioxidant effects stronger than vitamin C and E [15]. The Nrf2/ARE pathway is a significant antioxidant pathway. Nrf2 is released from Keap1 by ROS or through an electrophilic reaction. Thereafter, the compound then binds to the antioxidant response element (ARE) in the nucleus and mediates expression of peroxiredoxin and phase II detoxification enzymes (HO-1, GCLC, GCLM and NQO-1), which act as scavengers of ROS and protect cells from oxidative damage [16].

The embryonic zebrafish brain contains several clusters of dopaminergic, noradrenergic and adrenergic neurons in locations similar to those found in the adult brain [17,18,19]. By 3 days post-fertilization (dpf), dopaminergic neurons are observed in the ventral diencephalon, the pretectum, the locus coeruleus, the olfactory bulb and the retina [20]. Compared to experimental mice models, zebrafish models are associated with shorter experimental periods, lower cost and reduced complexity in gene manipulation [21]. Rat pheochromocytoma cells (PC12 cells) are very similar to neurons in morphology, structure and function [22]. They are highly consistent with primary cultured nerve cells, and have the advantages of easy and stable culture [23]. PC12 cells have been widely used as models for studying nerve cells [24,25,26].

The neurotoxin 1-methyl-4-phenyl-1,2,3,6-tetrahydropyridine (MPTP) has been shown to cause a syndrome in humans that resembles Parkinson’s disease. MPTP causes selective degeneration of dopaminergic neurons in the substantia nigra, and patients exhibit symptoms including tremors, immobility and a shuffling gait [27]. MPTP is metabolized to 1-methyl-4-phenylpyridinium (MPP^+^) in glial cells in the brain. After release from the glia, MPP^+^ is transported into dopaminergic neurons via the dopamine transporter (DAT) [28] and accumulates in the mitochondria, where it is believed to cause cell death by disrupting respiratory enzymes and causing oxidative damage [29,30]. Previous studies have found that PCs prevent some aging processes of female rats [31] and have certain protective effects on nerves [32,33]. However, whether PCs can prevent or reverse neuronal degeneration in PD by regulating the Nrf2/ARE pathway remains unclear. This study employed MPP^+^-treated PC12 cells [34,35] and MPTP-treated zebrafish [36] to assess the protective effects and potential mechanism of PCs on a model of PD.

## 2. Results

### 2.1. Effects of PCs on MPP^+^-Induced Cytotoxicity of PC12 Cells

**Figure 1:**
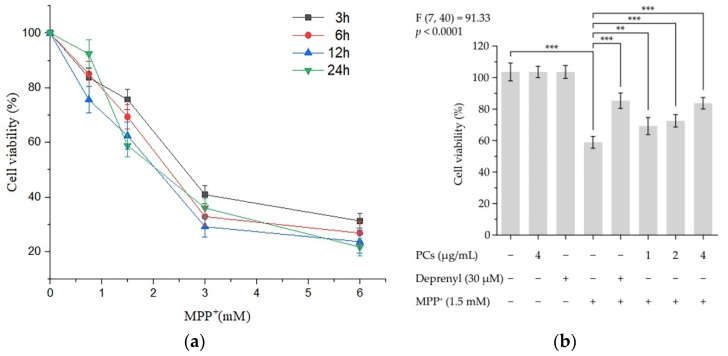
PCs on MPP^+^-induced cytotoxicity of PC12 cells. Cell viability was detected by CCK-8 assays. (**a**) Cytotoxic effects of MPP^+^ at different concentrations in PC12 cells; (**b**) PC-attenuated MPP^+^ induced decreases in cell viability. F and *p* values of the one-way analysis of variance are presented above the chart. The results of Tukey’s post hoc test are presented for selected comparisons: **, *p* < 0.01; ***, *p* < 0.001; the error bars are standard deviation (SD).

Incubation of PC12 cells to 1.5 mM MPP^+^ for 24 h resulted in cell viability of 58.88% (Figure 1a). Pretreatment of cells with PCs protected PC12 cells from MPP^+^-induced damage by increasing the cell viability (Figure 1b). There was no marked difference in cell survival rate between the 4 μg/mL PCs group and 30 μM deprenyl group (positive control group) (Figure 1b).

### 2.2. PCs Reduced MPP^+^-Induced Oxidative Stress and Increased Antioxidant Enzyme Activity

**Figure 2:**
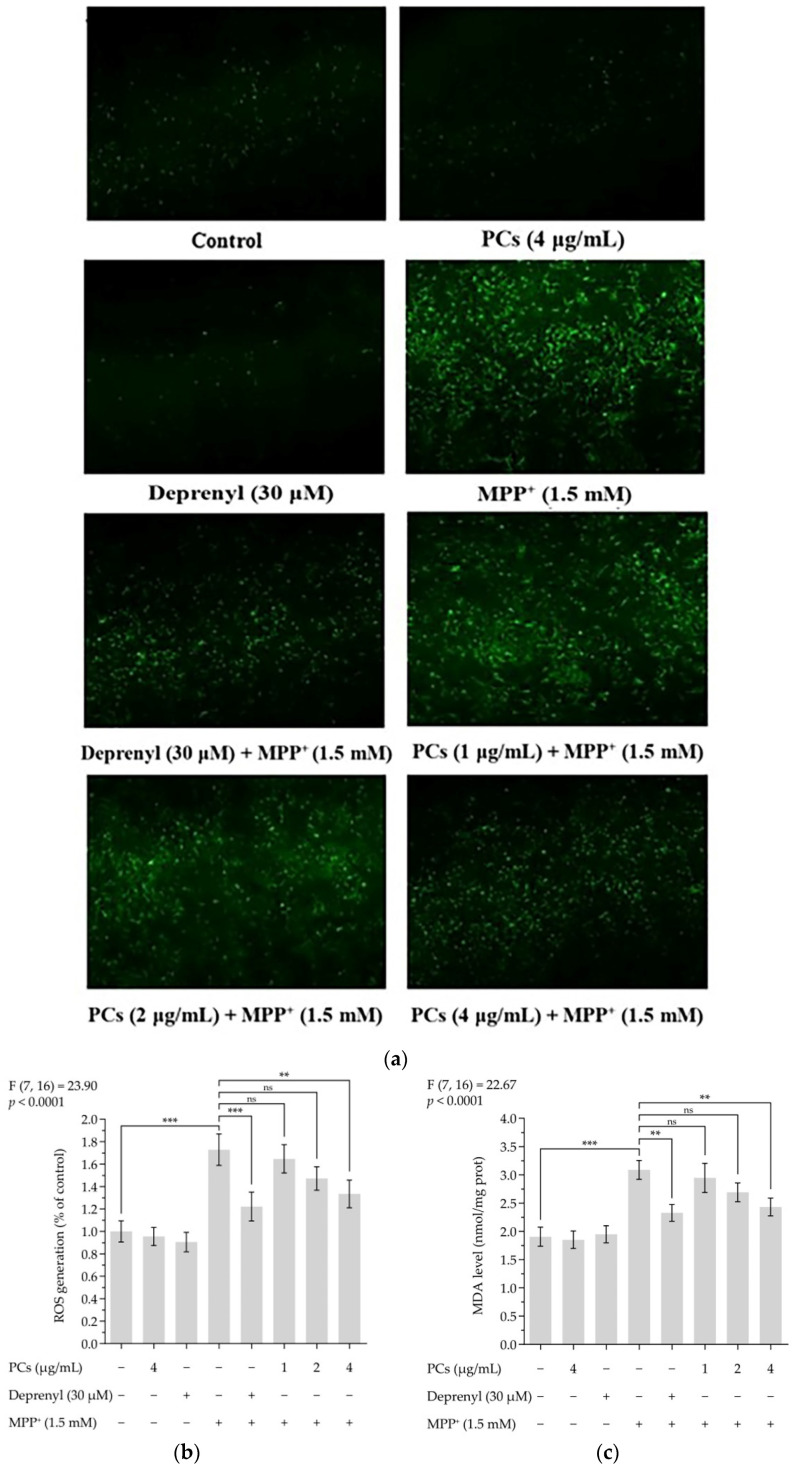

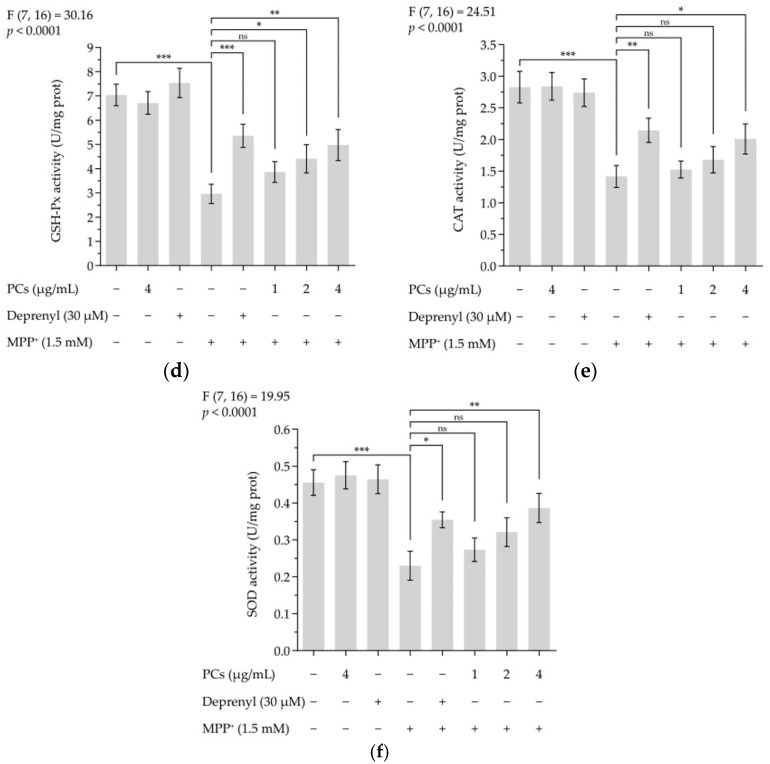
PCs reduced MPP^+^-induced oxidative stress and increased antioxidant enzyme activity. (**a**) ROS levels were measured by fluorescent microscopy and imaging analysis; (**b**) ROS levels were measured by image J software; (**c**) MDA levels; (**d**) GSH-Px activity; (**e**) CAT activity; (**f**) SOD activity. F and *p* values of the one-way analysis of variance are presented above each chart. The results of Tukey’s post hoc test are presented for selected comparisons: ns, *p* > 0.05; *, *p* < 0.05; **, *p* < 0.01; ***, *p* < 0.001; the error bars are SD.

ROS and malondialdehyde (MDA) levels indicate the severity of oxidative damage. Exposing PC12 cells to 1.5 mM MMP^+^ for 24 h increased ROS and MDA levels, which were inhibited by PCs at 4 μg/mL (Figure 2a–c). To further evaluate the effect of PCs on antioxidant capabilities of MPP^+^-treated PC12 cells, we measured glutathione peroxidase (GSH-Px), catalase (CAT), and superoxide dismutase (SOD) activity and observed that MPP^+^ treatment suppressed GSH-Px, CAT, and SOD activity. However, these MPP^+^ effects were rescued by PCs exposure at 4 μg/mL (Figure 2d–f).

### 2.3. Effects of PCs on Nrf2/ARE Pathway in MPP^+^-Induced PC12 Cells

**Figure 3:**
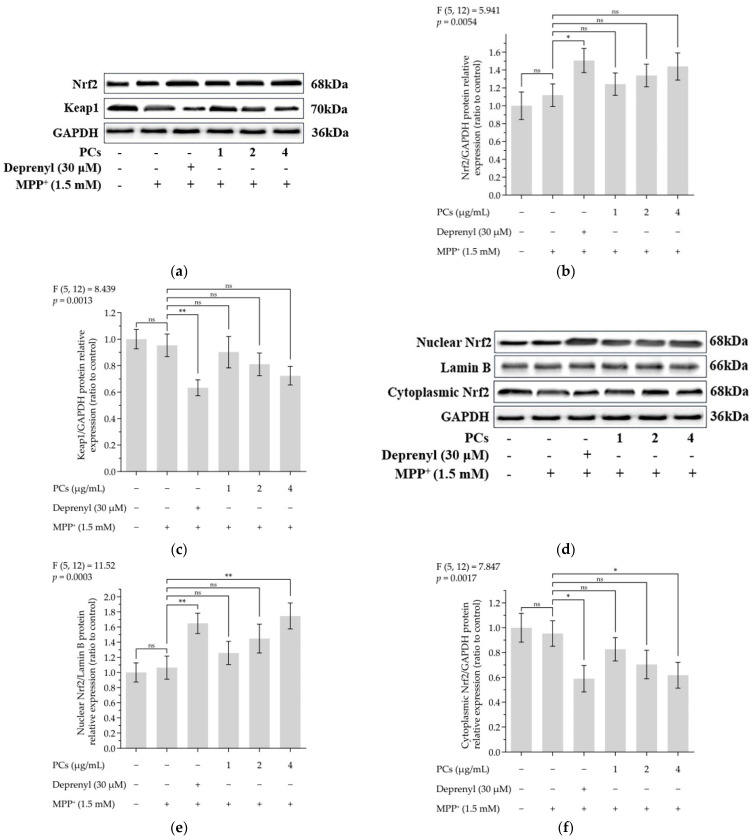

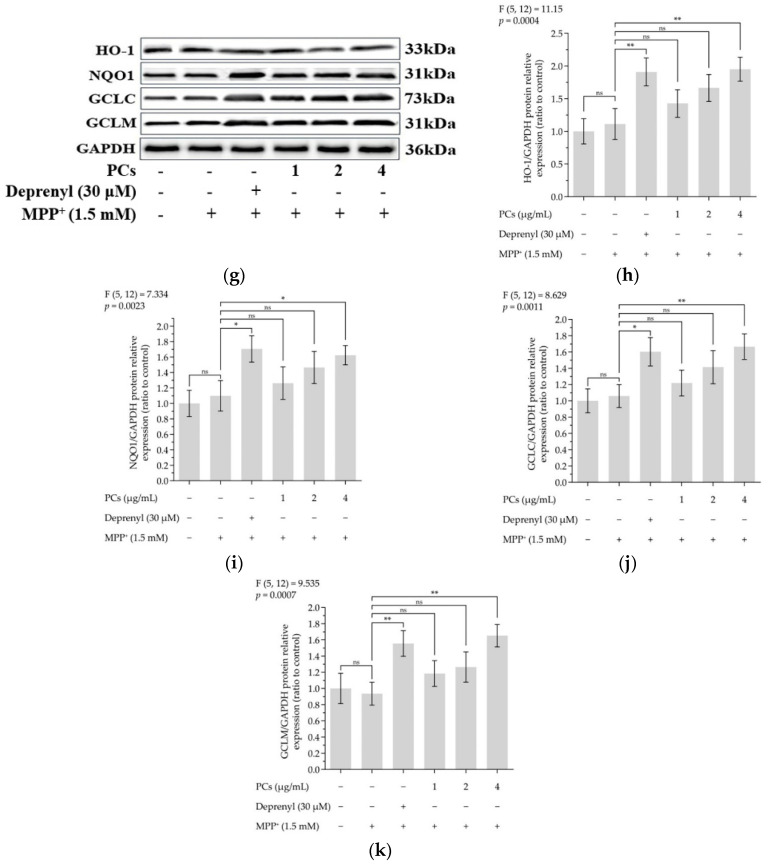
Effects of PCs on Nrf2/ARE pathway in MPP^+^-induced PC12 cells. (**a**) Protein levels of Nrf2 and Keap-1, as determined by Western blotting; (**b**) Nrf2/GAPDH protein relative expression (ratio to control); (**c**) Keap1/GAPDH protein relative expression (ratio to control); (**d**) protein expression levels of nuclear Nrf2 and cytoplasmic Nrf2, as determined by Western blotting; (**e**) nuclear Nrf2/LaminB protein relative expression (ratio to control); (**f**) cytoplasmic Nrf2/GAPDH protein relative expression (ratio to control); (**g**) protein levels of HO-1, NQO1, GCLC and GCLM, as determined by Western blotting; (**h**) HO-1/GAPDH protein relative expression (ratio to control); (**i**) NQO1/GAPDH protein relative expression (ratio to control); (**j**) GCLC/GAPDH protein relative expression (ratio to control); (**k**) GCLM/GAPDH protein relative expression (ratio to control). F and *p* values of the one-way analysis of variance are presented above each chart. The results of Tukey’s post hoc test are presented for selected comparisons: ns, *p* > 0.05; *, *p* < 0.05; **, *p* < 0.01; the error bars are SD.

The expression of nuclear factor-erythroid 2-related factor 2 (Nrf2) was upregulated, and the level of Keap1 was downregulated following PC treatment (Figure 3a–c). PCs (4 μg/mL) facilitated nuclear localization of Nrf2 under MPP^+^ lesion conditions (Figure 3d–f). Additionally, Western blot data showed a marked upregulation in the expression of heme oxygenase 1 (HO-1), quinone oxidoreductase 1 (NQO1), glutamate-cysteine ligase catalytic subunit (GCLC) and glutamate-cysteine ligase modifier subunit (GCLM) expression after PC (4 μg/mL) treatment, compared with MPP^+^ exposure alone (Figure 3g–k).

### 2.4. Nrf2/ARE Signaling Is Related to the Neuroprotective and Antioxidant Effects Mediated by PCs

**Figure 4:**
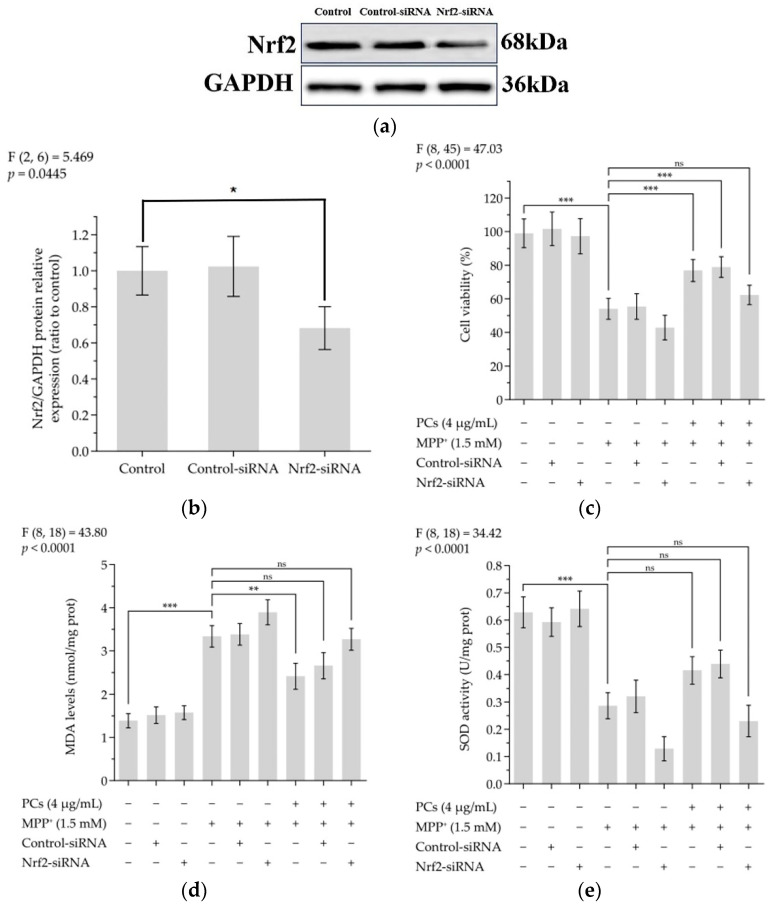
Nrf2/ARE signaling is related to the neuroprotective and antioxidant effects mediated by PCs. (**a**) Knockout efficiency was detected by determination of Nrf2 protein expression using Western blotting; (**b**) Nrf2/GAPDH protein relative expression (ratio to control); (**c**) Cell viability; (**d**) MDA levels; (**e**) SOD activity. F and *p* values of the one-way analysis of variance are presented above each chart. The results of Tukey’s post hoc test are presented for selected comparisons: ns, *p* > 0.05; *, *p* < 0.05; **, *p* < 0.01; ***, *p* < 0.001; the error bars are SD.

The knockdown of Nrf2 by siRNA effectively abolished the expression of Nrf2 (Figure 4a,b). Indeed, Nrf2 siRNA treatment abrogated the protective actions of PCs against MPP^+^-treated oxidative damage, as evidenced by the reduced cell viability in the Nrf2 siRNA-transfected group (Figure 4c). In addition, the effects of PCs on MDA and SOD were reversed by Nrf2 knockdown under MPP^+^ lesioning conditions (Figure 4d,e).

### 2.5. Effects of PCs on Zebrafish Larvae Motility upon MPTP Treatment

**Figure 5:**
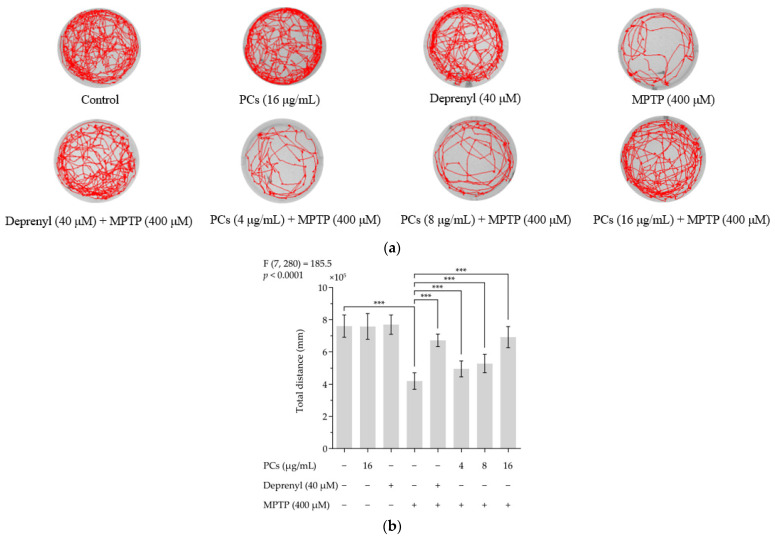
Effects of PCs on zebrafish larvae motility upon MPTP treatment. (**a**) Typical patterns of swimming traces of zebrafish larvae in each group; (**b**) Average total distance of zebrafish larvae in each group. F and *p* values of the one-way analysis of variance are presented above the chart. The results of Tukey’s post hoc test are presented for selected comparisons: ***, *p* < 0.001; the error bars are SD.

MPTP reduced the total distance zebrafish swam. PC (4, 8 and 16 μg/mL) treatment rescued MPTP-induced locomotive deficits and increased total distance traveled relative to the MPTP group (Figure 5a,b). Furthermore, deprenyl appeared to offer protection from MPTP-induced damage and was used as a positive control. Notably, PCs (16 μg/mL) and deprenyl (40 μM) alone did not affect locomotion behavior of normal zebrafish larvae.

### 2.6. Effects of PCs on MPTP-Induced Dopaminergic Neuron Injury in Zebrafish

**Figure 6:**
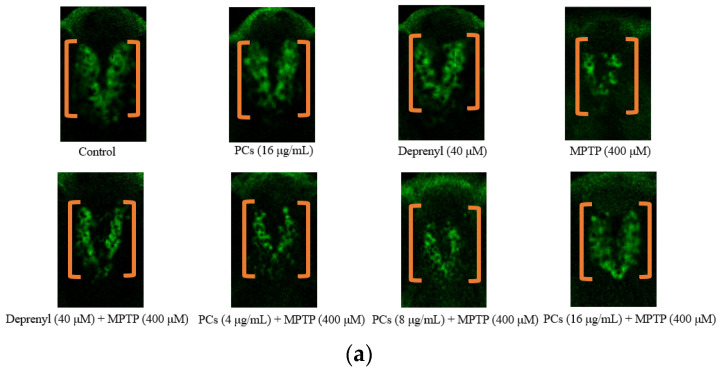

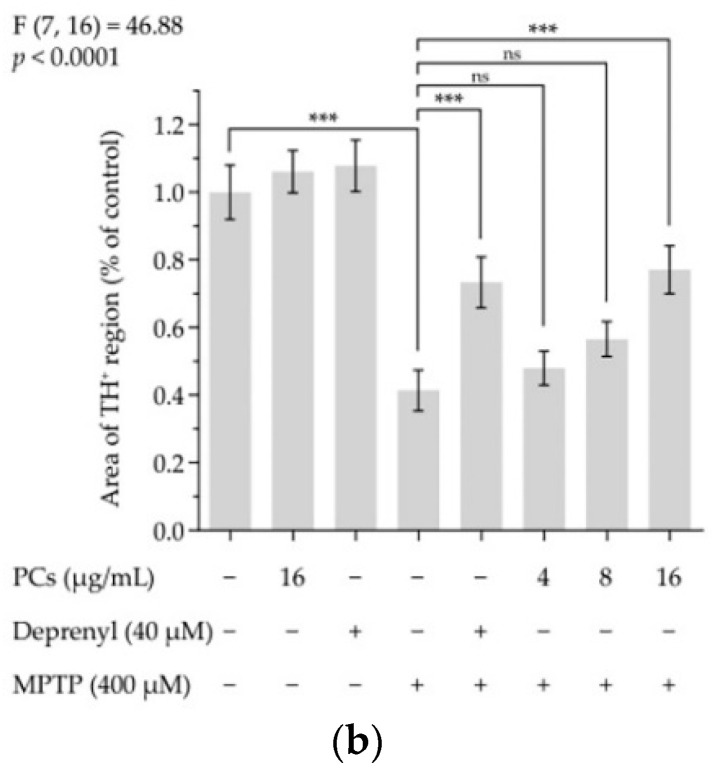
Effects of PCs on MPTP-induced dopaminergic neuron injury in zebrafish. (**a**) Representative pictures of the dopaminergic neurons in the brains of zebrafish; (**b**) number of TH^+^ neurons was measured by ImageJ. F and *p* values of the one-way analysis of variance are presented above the chart. The results of Tukey’s post hoc test are presented for selected comparisons on the chart: ns, *p* > 0.05; ***, *p* < 0.001; the error bars are SD.

Zebrafish embryos incubated with MPTP demonstrated a statistically significant decrease in tyrosine hydroxylase (TH) density (Figure 6a,b). Nevertheless, treatment with 16 μg/mL of PCs significantly reversed the reduction in TH^+^ cell density (Figure 6a,b).

### 2.7. Effects of PCs on Oxidative Stress of Zebrafish Larvae Treated with MPTP

**Figure 7:**
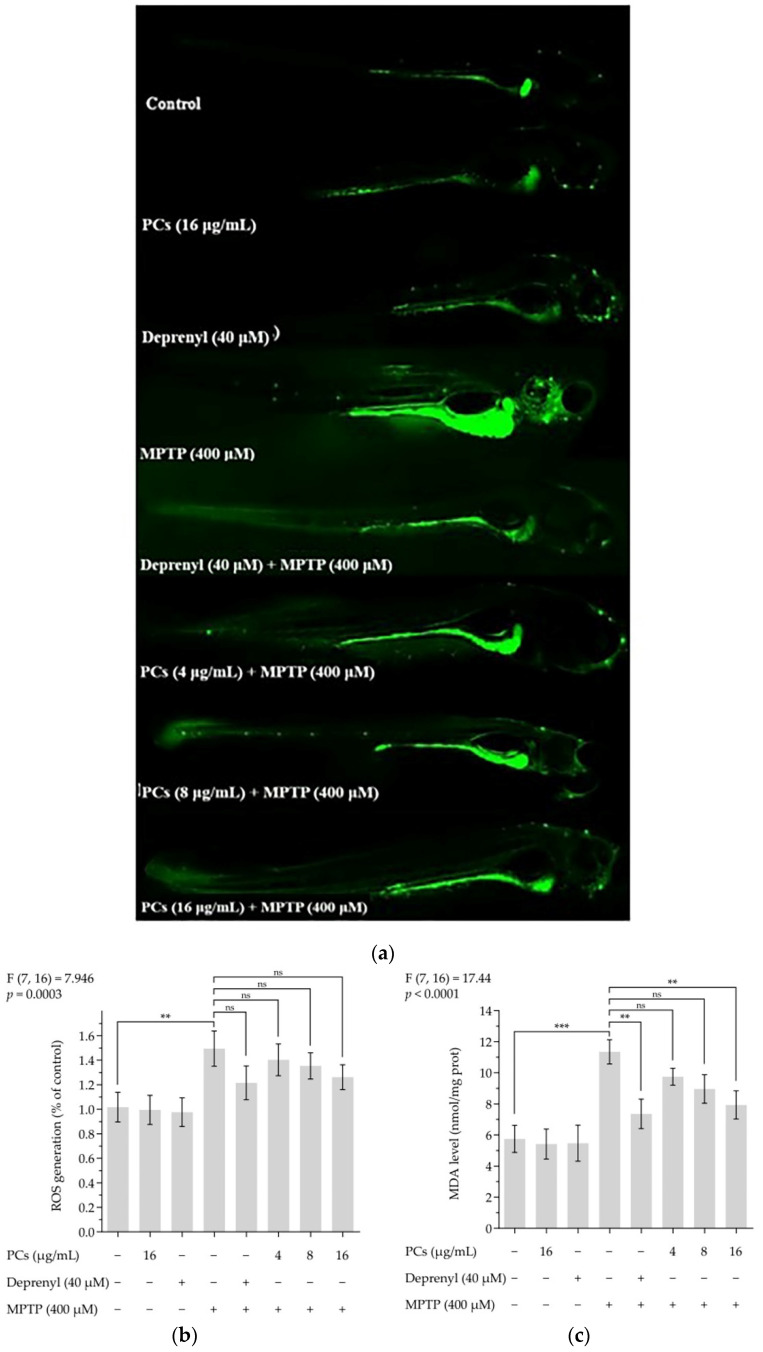

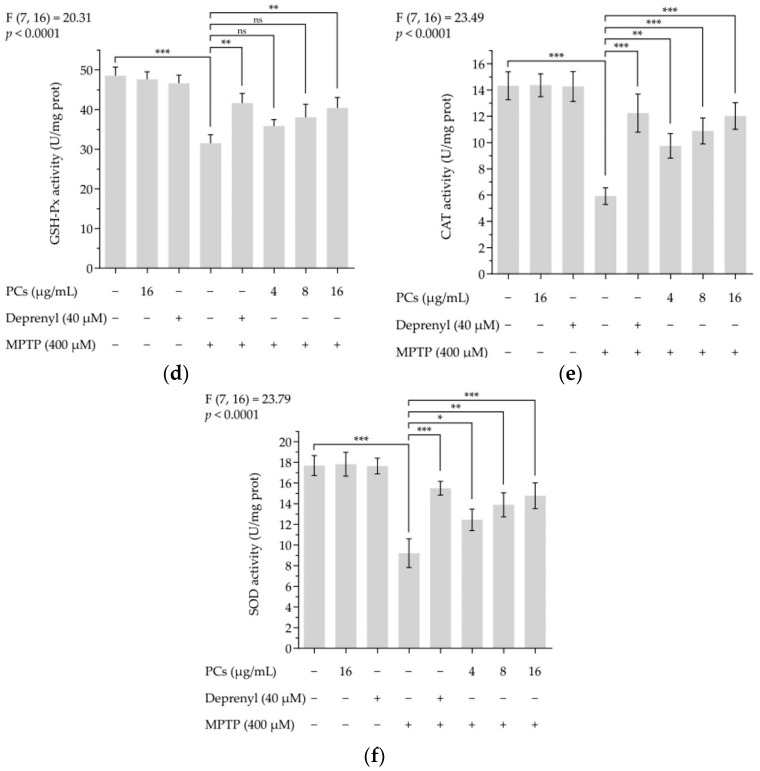
Effects of PCs on oxidative stress of zebrafish larvae treated with MPTP. (**a**) ROS levels were measured by fluorescent microscopy and imaging analysis; (**b**) ROS levels were measured via image J software; (**c**) MDA levels; (**d**) GSH-Px activity; (**e**) CAT activity; (**f**) SOD activity. F and *p* values of the one-way analysis of variance are presented above each chart. The results of Tukey’s post hoc test are presented for selected comparisons: ns, *p* > 0.05; *, *p* < 0.05; **, *p* < 0.01; ***, *p* < 0.001; the error bars are SD.

Treatment of zebrafish larvae with PCs decreased MPTP-induced increased intracellular ROS formation (Figure 7a,b). Furthermore, the lipid peroxidation assay results showed that PC exposure (16 μg/mL) blocked the MPTP-induced MDA levels in zebrafish larvae (Figure 7c). Additionally, the MPTP-induced reduction in GSH-Px activity was reversed by PC (16 μg/mL) treatment (Figure 7d), and PCs (4, 8, and 16 μg/mL) increased MPTP-induced decreases in CAT and SOD activity (Figure 7e,f).

### 2.8. Effects of PCs on Nrf2/ARE Pathway in MPTP-Induced Zebrafish Larvae

**Figure 8:**
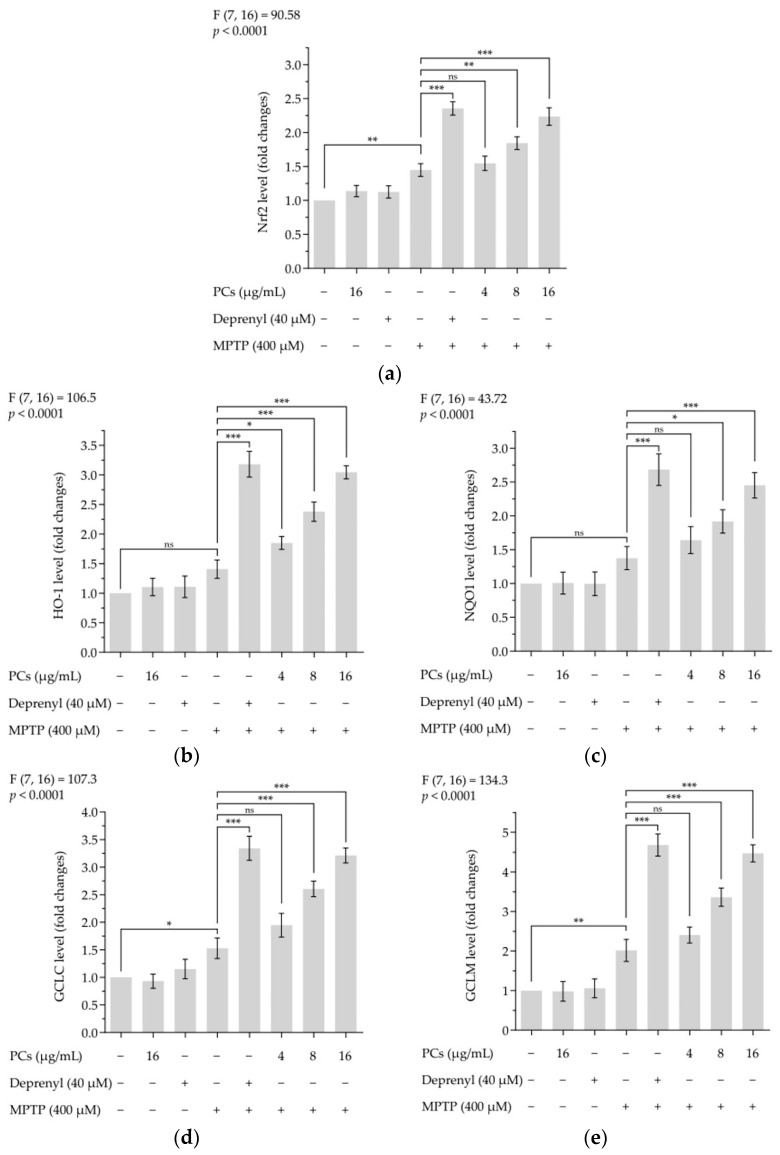
Effects of PCs on Nrf2/ARE pathway in MPTP-induced zebrafish larvae. (**a**) Nrf2 levels; (**b**) HO-1 levels; (**c**) NQO1 levels; (**d**) GCLC levels; (**e**) GCLM levels. F and *p* values of the one-way analysis of variance are presented above each chart. The results of Tukey’s post hoc test are presented for selected comparisons: ns, *p* > 0.05; *, *p* < 0.05; **, *p* < 0.01; ***, *p* < 0.001; the error bars are SD.

Treatment of zebrafish larvae with PCs underscored the premise that activation of Nrf2/ARE pathways was involved in PC-mediated protective properties. The expression of Nrf2, HO-1, NQO1, GCLC and GCLM markers was upregulated by PCs treatment compared with MPTP exposure alone (Figure 8a–e).

## 3. Discussion

PCs exhibit strong radical scavenging abilities and antioxidant activity, and oxidative stress, caused by the excessive generation of ROS or/and the impaired antioxidant defense system, plays a critical role in PD [37,38,39]. However, whether PCs can play a neuroprotective role through antioxidant effects remains unclear. This research aimed to investigate the molecular mechanism of PCs against MPP+/MPTP-induced PD models.

In this study, compared with the MPP^+^/MPTP-alone group, PCs markedly raised the activity level of antioxidant enzymes (including GSH-Px, CAT and SOD) and decreased levels of ROS and MDA. The current findings are consistent with a previous study that suggests that a low PC supplement via diet improves the activities of GSH-Px, CAT and SOD in weaned piglets [40]. Another study also suggests that PCs significantly increase CAT and SOD activities and decrease MDA content, thus improving the quality of goat sperm [41]. These findings suggest that PCs have a protective effect on oxidative damage of nerve cells. Additionally, PCs improved cell viability compared with the MPP^+^-alone group in MPP^+^-induced PC12 cells, markedly increased total distance moved and decreased TH^+^ cell density relative to the MPTP group in MPTP-induced zebrafish larvae. These data indicate that PCs have a protective effect on nerve cells.

We further observed that PCs significantly increased nuclear Nrf2 accumulation compared with that of MPP^+^ alone in PC12 cells. Indeed, PCs markedly upregulated the expression levels of NQO1, HO-1, GCLM and GCLC [42]. Furthermore, Nfr2 gene silencing via Nrf2 siRNA was used to investigate the role of Nrf2/ARE activation in PCs-mediated neuroprotection against MPP^+^-induced oxidative damage: Nrf2-siRNA-transfected cells indicated a marked decrease in Nrf2 expression. This current study also found that Nrf2 knockout abolished both PCs-mediated protection against MPP^+^-treated impairments in cell viability and the antioxidant effects of PCs. These results are consistent with a previous study suggesting that improving activation of the Nrf2/ARE pathway contributes to neuroprotection [43].

The Nrf2/ARE pathway is a significant antioxidant pathway [44,45,46]. Normally, Nrf2 resides in the cytoplasm, where it is bound to the inhibitory protein, Keap1 [47,48]. When cells undergo oxidative stress, Nrf2 dissociates from Keap1, initiates the endogenous antioxidant defense system and subsequently translocates into the nucleus [46,49,50]. It then interacts with ARE to activate a series of cell-protective and antioxidant genes, including GCLC, GCLM, NQO1 and HO-1 [51,52,53,54]. In response to oxidative stress, Nrf2 dissociates from Keap1 in the cytosol and is then translocated into the nucleus, binding to the ARE sequence to activate transcription of cryoprotective genes [53,54,55,56,57,58]. The current results indicate that PCs can activate the Nrf2/ARE pathway, transfer Nrf2 from the cytoplasm to the nucleus, accumulate in the nucleus, upregulate the expression of GCLC, GCLM, NQO1 and HO-1 and improve the ability of cells to resist oxidative stress. Indeed, the Nrf2/ARE pathway may represent a pharmacological target of PCs for the prevention of PD.

## 4. Materials and Methods

### 4.1. Chemical Compounds and Reagents

MPP^+^ and MPTP agents were purchased from Sigma (St. Louis, MO, USA), and the Cell Counting Kit-8 was obtained from Beyotime (Shanghai, China). The PCs, 2′,7′-Dichlorofluorescin diacetate, MDA, GSH-Px, SOD and CAT diagnostic kits were all obtained from Solarbio (Beijing, China). The RNAiso Plus, PrimeScript™RT Reagent Kit with gDNA Eraser, and SYBR^®^Premix Ex Taq™ II were purchased from Takara (Shiga, Japan); the Nrf2-siRNA, control-siRNA, and Lipofectamine 2000 were obtained from GenePharma (Shanghai, China). Finally, the primary antibodies, Nrf2, HO-1, GCLC, GCLM, NAD(P)H: NQO1, Lamin B, GAPDH, Antityrosine Hydroxylase (TH) and corresponding secondary antibodies were obtained from Proteintech (Wuhan, China).

### 4.2. Cell Culture

PC12 cells were obtained from the National Collection of Authenticated Cell Cultures. The cells were maintained in DMEM supplemented with 10% fetal bovine serum and penicillin-streptomycin (100 U/mL; 100 μg/mL) in a humidified atmosphere incubator at 37 °C with 5% CO_2_ [59].

### 4.3. Cell Viability Assay

PC12 cells (1 × 10^4^ cells/well) were incubated with 1, 2 or 4 μg/mL of PCs or deprenyl (30 μM) for 24 h, and then incubated with 1.5 mM MPP^+^ for 24 h. Next, 10 μL of Cell Counting Kit-8 solution was added into each well and incubated for 1 h. The absorbance was measured at 450 nm [60].

### 4.4. Fish Maintenance

Ethical approval for animal use was granted by the animal conservation and use committee of the experimental animal center, Zhejiang University (ZJU20200125). Adult zebrafish (AB strain) were obtained from the laboratory animal center of Zhejiang University (Hang Zhou, China) and maintained at 28 ± 1 °C under 14 h light/10 h dark cycles. The fish were fed *Artemia nauplii* twice daily [61]. To produce embryos, adult zebrafish were placed in breeding tanks overnight at a 1:1 male:female ratio. Spawning was triggered after the lights were turned on the next morning and completed in 2 h. Embryos were raised in embryo water (13.7 mM NaCl, 540 μM KCl, 25 μM Na2HPO_4_, 44 μM KH_2_PO_4_, 300 μM CaCl_2_, 100 μM MgSO_4_, 420 μM NaHCO_3_, pH 7.4) at 28 °C [62,63].

### 4.5. ROS Measurement

To measure ROS production, PC12 cells (2 × 10^4^ cells/well) were exposed to 1, 2 or 4 μg/mL of PCs or deprenyl (30 μM) for 24 h, and then incubated with 1.5 mM MPP^†^ for 24 h [64]. The cells were exposed to 10 μM 2’,7’-Dichlorofluorescin diacetate solution in dark conditions for 30 min; the dye solution was then removed, and the cells were washed with phosphate-buffered saline (PBS) 3 times. The images of the cells were observed using an Olympus laser scanning confocal microscope (Olympus, Tokyo capital, Japan). The fluorescence intensity of the cells was quantified using Image J software v1.8.0 (Olympus, Tokyo capital, Japan). The results are expressed as a percentage of area of the ROS regions in the control group.

To measure ROS production, the zebrafish larvae at 3 days post fertilization (dpf) were exposed to deprenyl (40 μM) or 4, 8 or 16 μg/mL of PCs with or without 400 μM MPTP for 4 days. Zebrafish larvae at 7 dpf were transferred to a 24-well plate (10 larvae per group), treated with 20 μM 2’,7’-Dichlorofluorescin diacetate solution and incubated for 60 min in the dark at 28.5 °C [65]. The larvae were then washed three times with embryo medium to remove excess 2’,7’-Dichlorofluorescin diacetate. The images of the stained larvae were observed using an Olympus laser scanning confocal microscope. The fluorescence intensity of the individual larva was quantified using Image J software. The results are expressed as a percentage of area of the ROS regions in the control group.

### 4.6. Assessment of MDA, GSH-Px, SOD, and CAT

PC12 cells (2 × 10^4^ cells/well) were exposed to 1, 2 or 4 μg/mL of PCs or deprenyl (30 μM) for 24 h, and then incubated with 1.5 mM MPP^+^ for 24 h. Then, 1 mL of extract was added to 4 × 10^6^ cells and cells were broken by ultrasonic centrifuging at 8000 rpm at 4 °C for 10 min. The supernatant was then put on ice for testing, and reagents were added for the determination of MDA, GSH-Px, SOD and CAT, respectively. Each index was repeated in triplicate.

The zebrafish larvae at 3 dpf were incubated with deprenyl (40 μM) or 4, 8 or 16 μg/mL of PCs with or without 400 μM MPTP for 4 days. Then, 0.05 g of zebrafish larvae tissue and 0.5 mL of extract were homogenized in an ice bath and then centrifuged at 4 °C for 10 min. The supernatant was put on ice for testing. Reagents were subsequently added for the determination of MDA, GSH-Px, SOD and CAT, respectively. Each index was repeated in triplicate.

### 4.7. Preparation of Whole Cell, Cytoplasmic, and Nuclear Protein

For whole-cell protein extraction, cells were collected and incubated with RIPA lysis buffer containing 1% PMSF and 1% protease inhibitor cocktail for 30 min on ice. Cell lysates were centrifuged, and the supernatant was collected and stored. For subcellular fractionation preparation, cell samples were processed using the nuclear and cytoplasmic protein extraction kit. The protein content was assayed using the BCA (Beyotime, Shanghai, China) assay.

### 4.8. Nrf2 siRNA Transfection

Nrf2-siRNA was used to knock down Nrf2. PC12 cells were transfected with Nrf2-siRNA (80 nM) or control-siRNA using Lipofectamine 2000, according to the manual.

### 4.9. Western Blotting

Protein samples were resolved by SDS-PAGE and transferred to polyvinylidene difuoride (PVDF) membranes. The blots were exposed to appropriate primary antibodies: Nrf2, Keap1, HO-1, NQO1, GCLC, GCLM, Lamin B, GAPDH and peroxidase-conjugated secondary antibodies. Protein bands were visualized using ECL (Beyotime, Shanghai, China) plus Western blotting detection reagents [36,43].

### 4.10. Locomotor Behavioral Test

The zebrafish larvae at 3 dpf were incubated with deprenyl (40 μM) or 4, 8 or 16 μg/mL of PCs with or without 400 μM MPTP for 4 days. The 7 dpf zebrafish were then placed into 24-well plates (1 fish per well and 12 larvae per group), and the total distance each fish swam over 10 min was recorded. Zebrafish behavior was monitored and analyzed using an automated video tracking system (Any-maze 4.73, Stoelting, Wood Dale, IL, USA) [66].

### 4.11. Total RNA Extraction, Reverse Transcription, and Quantitative Real-Time Polymerase Chain Reaction

RNA was extracted using RNAiso Plus following the manufacturer’s instructions. RNA was reverse transcribed using a PrimeScript™RT Reagent Kit with gDNA Eraser, following the manufacturer’s instructions. Quantitative real-time PCR analysis was performed on an Applied Biosystems ViiA™ 7 Real-Time PCR system using SYBR^®^Premix Ex Taq™ II (Takara, Shiga, Japan). β-actin was used as a reference gene and relative gene expression was calculated using the 2^–ΔΔCt^ method. The primer sequences utilized in the research are listed in Table 1.

### 4.12. Zebrafish Antityrosine Hydroxylase (TH) Whole-Mount Immunostaining

Zebrafish larvae at 1 dpf were incubated with deprenyl (40 μM) or 4, 8 or 16 μg/mL of PCs with or without 400 μM MPTP for 2 days (10 fish/group batches). Larvae were fixed with 4% paraformaldehyde in PBS for 30 min. After fixation, they were treated for whole-mount immunostaining of TH [67]. At room temperature, 2% (*v*/*v)* lamb serum and 0.1% (*w*/*v*) bovine serum albumin (BSA) were blocked in phosphate-buffered saline Tween-20 (PBST) for 1 h. They were then exposed in the blocking buffer to antityrosine hydroxylase antibody (1:200 diluted, Proteintech) for 2 h, and then rinsed 6 times with PBST. The final whole-mount immunostaining step was performed in staining buffer with 488 goat antirabbit (1:500) for 1 h and washed again with PBST. After sufficient color development, the zebrafish were flat mounted with 3.5% methylcellulose and imaged using an Olympus laser scanning confocal microscope.

### 4.13. Statistical Analysis

One-way analysis of variance (ANOVA) followed by Tukey’s multiple comparison test were used to compare the means of different groups. Graphpad Prism v6.01 (GraphPad Software, San Diego, CA, USA) was used for statistical analysis and plotting the graphs.

## 5. Conclusions

In conclusion, our findings indicate that PCs exert neuroprotective effects via activation of the Nrf2/ARE pathway and its downstream detoxification and antioxidant enzymes. Taken together, these insights suggest that PCs may be useful for treating PD.

## Figures and Tables

**Table 1:** Sequences of primers for quantitative real-time PCR.

Genes	Forward Primer	Reverse Primer
β-Actin	CACTGAGGCTCCCCTGAATC	GGGTCACACCATCACCAGAG
Nrf2	CTGCTGTCACTCCCAGAGTT	GCCGTAGTTTTGGGTTGGTG
HO-1	AAGAGCTGGACAGAAACGCA	AGAAGTGCTCCAAGTCCTGC
GCLC	CTCCTCACAGTCACGGCATT	TGAATGGAGACGGGGTGTTG
GCLM	AAGCCAGACACTGACACACC	ATCTGGAGGCATCACACAGC
NQO1	AAGCCTCTGTCCTTTGCTCC	TGCTGTGGTAATGCCGTAGG

Nrf2, nuclear factor-erythroid 2-related factor 2; HO-1, heme oxygenase 1; GCLC, glutamate-cysteine ligase catalytic subunit; GCLM, glutamate-cysteine ligase modifier subunit; NQO1, quinone oxidoreductase 1.

## Data Availability

Data is contained within the article.

## Sample Availability

Samples of the compounds are not available from the authors.

## Abbreviations

PCs: procyanidins; MPTP: 1-methyl-4-phenyl-1,2,3,6-tetrahydropyridine; MPP^+^: 1-methyl-4-phenylpyridinium; PD: Parkinson’s disease; dpf: days post-fertilization; GSH-Px: glutathione peroxidase; SOD: superoxide dismutase; CAT: catalase; ROS: reactive oxygen species; MDA: malondialdehyde; Nrf2: nuclear factor-erythroid 2-related factor 2; ARE: antioxidant response element; HO-1: heme oxygenase 1; NQO1: quinone oxidoreductase 1; GCLC: glutamate-cysteine ligase catalytic subunit; GCLM: glutamate-cysteine ligase modifier subunit; TH: tyrosine hydroxylase; SD: standard deviation; ANOVA: one-way analysis of variance.
